# A meta-analysis of the clinical significance of neutrophil-to-lymphocyte ratios in interstitial lung disease

**DOI:** 10.1371/journal.pone.0286956

**Published:** 2023-06-12

**Authors:** Fei Dong, Leting Zheng, Weiwei An, Ting Xue, Xiaoning Zhong

**Affiliations:** 1 Respiratory and Critical Care Medicine Department, The First Affiliated Hospital of Guangxi Medical University, Nanning, Guangxi Zhuang Autonomous Region, China; 2 Rheumatology and Immunology Department, The First Affiliated Hospital of Guangxi Medical University, Nanning, Guangxi Zhuang Autonomous Region, China; Osaka University of Pharmaceutical Sciences, JAPAN

## Abstract

Interstitial lung disease (ILD) is a group of diffuse parenchymal infiltrating diseases of different etiologies. The neutrophil-to-lymphocyte ratio (NLR) can reflect ILD’s existence, progression, and prognosis and is currently regarded as a promising biological marker. This meta-analysis assessed elevated NLR levels in ILD for their predictive value. From inception to July 27, 2022, the Scopus, Cochrane Library, Web of Science, Embase, and PubMed databases were checked thoroughly. We used the weighted mean difference (WMD) and 95% confidence interval (CI) to compare blood NLR values between groups. We examined the relationship between poor prognoses and elevated NLR concentrations in ILD patients using odds ratios (ORs) and 95% CI. After initially including 443 studies, 24 were ultimately analyzed. Fifteen studies(ILD:n = 2,912, Non-ILD: n = 2,868) revealed that the NLR values in the ILD group were relatively high (WMD = 0.61, 95% CI 0.43–0.79, *p* = 0.001). Eight articles (with poor prognoses: n = 407, without poor prognoses: n = 340) indicated that ILD patients with poor prognoses had higher NLR values (WMD = 1.33, 95% CI 0.32–2.33, *p* = 0.01). This distinction was especially noticeable in patients with the connective tissue disease (CTD)associated with ILD subgroup (WMD = 3.53, 95% CI 1.54–5.51, *p* = 0.0005). The pooled OR for increased NLR levels forecasting poor prognoses of ILD was 1.09 (95% CI 1.03–1.15, *p* = 0.0008). Increasing blood NLR values have clinical significance and application value for detecting ILD and predicting its poor prognosis, especially in CTD patients.

## Introduction

Interstitial lung disease (ILD) is a group of diffuse pulmonary ailments caused by varying known or unknown causes, with different degrees of inflammation, fibrosis, and lung parenchyma damage. It is a refractory respiratory disease that affects the pulmonary interstitium, bronchioles, and/or alveoli. The early manifestation is alveolitis, and lung function gradually deteriorates, eventually leading to pulmonary fibrosis.

ILD poses a significant public health problem, accounting for 0.26% of all-cause mortality in 2017 [1]. It has an annual incidence of 1–31.5 per 100,000 people and a prevalence of 6.3–71 per 100,000 [2]. According to a Global Burden of Disease Study, the global age-standardized fatality rate from pulmonary interstitial fibrosis and pulmonary sarcoidosis rose by 0.97% (0.92% to 1.03%) annually from 1990 to 2017 [1]. Over the past 30 years, ILD has shown increasing trends in global incidence, deaths, and disability-adjusted life years.

Self-sustaining gradual fibrosis in the pulmonary parenchyma worsens respiratory ailments, pulmonary function, and quality of life. After diagnosis, ILD usually has a poor prognosis due to the lack of effective treatments [3]. The five-year survival rate for ILD patients is just 56% [4]. Accurate diagnosis of ILD is challenging and necessitates a multidisciplinary approach. Pulmonary function, radiological, and histopathological examinations are currently the primary methods for diagnosing and assessing the severity of ILD and guiding treatment. Their invasiveness, difficulty in implementation, and limitations also make diagnosis difficult. Compared with the primary diagnostic methods, serum markers are easy to obtain, low-cost, reproducible, and less invasive, and they avoid lung biopsy. The neutrophil-to-lymphocyte ratio (NLR) derives from the ratio of neutrophils to lymphocytes. Recent research has found it is a new inflammatory biomarker linked to some diseases’ occurrence, progression, and prognosis, including inflammatory diseases and cancer [5]. Recent research has found a connection between increased blood NLR levels and the occurrence and progression of ILD, lung function decline, imaging progression, and all-cause mortality [6–22]. These results imply that NLR could be utilized as a novel inflammatory marker for ILD, predicting disease onset and progression. There has, however, been no systematic review of the relationship between blood NLR value and the presence, progression, and poor prognosis of ILD. This article used a meta-analysis to assess the utility of NLR in the clinical diagnosis of ILD and disease follow-up through a quantitative comparison between different groups.

## Methods

This meta-analysis was conducted under the Preferred Reporting Items for Systematic Reviews and Meta-analyses (PRISMA) 2020 statement and registered on the International Prospective Register of Systematic Reviews (PROSPERO, CRD42022349976).

### Data sources and search strategy

Two investigators (FD and LZ) independently searched the literature for all published observational studies on NLR and ILD in the following databases: Embase, the Cochrane Library, Web of Science, PubMed, and Scopus. The search period lasted from the database’s creation to July 27, 2022. We created search queries by integrating medical subject headings (MeSH) and free text. We tailored them to the characteristics of each database. The search strategy for Embase was the following: (’lung diseases, interstitial’/exp OR ’lung diseases, interstitial’ OR ’interstitial pneumonia’ OR ’interstitial pneumonias’ OR ’pneumonias, interstitial’ OR ’pneumonitis, interstitial’ OR ’interstitial pneumonitides’ OR ’interstitial pneumonitis’/exp OR ’diffuse parenchymal lung disease’ OR ’interstitial lung diseases’/exp OR ’interstitial lung diseases’ OR ’diffuse parenchymal lung diseases’ OR ’interstitial lung disease’/exp OR ’interstitial lung disease’ OR ’lung disease, interstitial’/exp OR ’lung disease, interstitial’ OR ’pneumonia interstitial’ OR ’interstitial pneumonia’/exp OR ’interstitial pneumonitis’ OR ’diffuse parenchymal lung disease’/exp OR ’pneumonitides, interstitial’:ab,kw,ti) AND (’neutrophil-to-lymphocyte ratio’/exp OR ’nlr’ OR ’neutrophil to lymphocyte ratio’/exp OR ’neutrophil to lymphocyte ratio’ OR ’neutrophil/lymphocyte ratio’/exp OR ’neutrophil-to-lymphocyte ratio’ OR ’neutrophil/lymphocyte ratio’ OR ’neutrophil-lymphocyte ratio’/exp OR ’neutrophil-lymphocyte ratio’ OR ’neutrophil lymphocyte ratio’:ab,kw,ti).

### Inclusion and exclusion criteria

The following were the inclusion criteria: (1) the work was a prospective or retrospective observational study; (2)the diagnosis of ILD was based on high-resolution computed tomography (HRCT), which showed typical imaging features of ILD, consistent with interstitial changes (nodular shadows, ground-glass shadows or patchy infiltrates, traction bronchiectasis, and tube wall thickening, interlobular septal thickening or irregular lines). Progression of ILD was defined as meeting at least 1 of the following criteria within one year: relative decrease in forced vital capacity(FVC) expected ≥ 10%; progressive dyspnoea and hypoxemia; and increased fibrosis on chest HRCT [3]; (3) NLR was measured as mean ± standard deviation (SD) or could be calculated in this manner. Studies were excluded if they featured (1) duplicate data or (2) participants under the age of 18, or were (3) animal studies, or (4) non-English publications. Furthermore, the most recent or complete study was chosen when the participants overlapped in multiple studies. There were two primary outcomes: (1) the discrepancy in NLR between patients with ILD and patients without ILD, and (2) the discrepancy in NLR between ILD patients who had unfavorable outcomes and those who had favorable outcomes.

### Data extraction and study selection

Two independent investigators (FD and LZ) reviewed the research and extracted and cross-checked the data. The two reviewers discussed the content of the discrepancies. If the disagreements remained unresolved, they would seek a third author’s advice (XZ) until reaching a consensus. When possible, missing data were supplemented by contacting the authors. When screening the literature, the titles and abstracts were read first, and then we proceeded after excluding irrelevant literature. We read the full texts carefully to determine the final inclusions. We extracted the following items from each study: ① basic study details, including the research topic, first researcher, publishing year, region, and research design type; ② baseline subject features, such as the source of every group, sample size, mean age, gender, and adverse outcomes; ③ major components of risk of bias evaluation; ④ important outcome predictors and assessments, including changes in NLR value (mean ± [SD]), adjusted odds ratio (OR), relative risk (RR), or hazard ratio (HR), with 95% confidence intervals (CI) for ILD-related adverse outcomes. We calculated the mean ± SD value in the study by Luo (2018) and Wan (2014) [23, 24], as the researchers provided only the number of participants and median and interquartile range of NLR.

### Risk of bias (Quality) assessment

We utilized the modified Newcastle-Ottawa Quality Assessment Scale (NOS) to examine the methodological quality of the cohort and case-control research. The NOS comprises patient selection, study group comparability, and outcome evaluation. Each publication was graded on a scale of 0 to 9 (allocated as stars), with seven or more stars suggesting a low possibility of bias. We evaluated the methodological quality of cross-sectional studies using the Agency for Healthcare Research and Quality (AHRQ). This instrument has 11 questions that can be answered with "Yes," "No," or "Unclear." If a response was given as “No” or “Unclear,” the item received a “0.” In this study, a rate of 8–11 was seen as high quality, a rate of 4–7 was regarded as moderate quality, and a rate of 0–3 was deemed low quality.

### Statistical analysis

We described the discrepancy in the NLR between the two groups using the weighted mean difference (WMD) and 95% confidence intervals (95% CI). We used OR and 95% CI (the test level was α = 0.05) to assess the link between elevated NLR and the poor prognosis of ILD patients. The HR/RR/OR values provided in the original literature were transformed using the standard inverse variance method and used to merge the logarithm values and standard errors. Associations between NLR and ILD presence and unfavorable outcomes were investigated. The heterogeneity of WMD values across the research was assessed using Cochran’s Q test (the test level was *p* < 0.10) [25]. The studies’ inconsistency was calculated using the I^2^ statistic: I^2^ = 0–25% was seen as no heterogeneity; I^2^ = 25%–50% was regarded as mild heterogeneity; I^2^ = 50%–75% was deemed moderate heterogeneity; I^2^ = 75% –100% was interpreted as extreme heterogeneity. Random-effect models were used with significant heterogeneity (I^2^ > 50%). Otherwise, a fixed-effects model would be used [26]. Furthermore, we calculated meta-regression and subgroup analyses to look for potential moderators. We employed sensitivity analysis to examine the heterogeneity sources by excluding studies sequentially. When more than ten articles were included, the Begg and Egger test was used to analyze the relationship between study size and effect magnitude. The *p*-value was set to a significance level of less than 0.05 [27, 28]. We used Stata (version 12.0) and RevMan5.3 for the statistical analyses.

## Results

### Study selection

**Fig 1:**
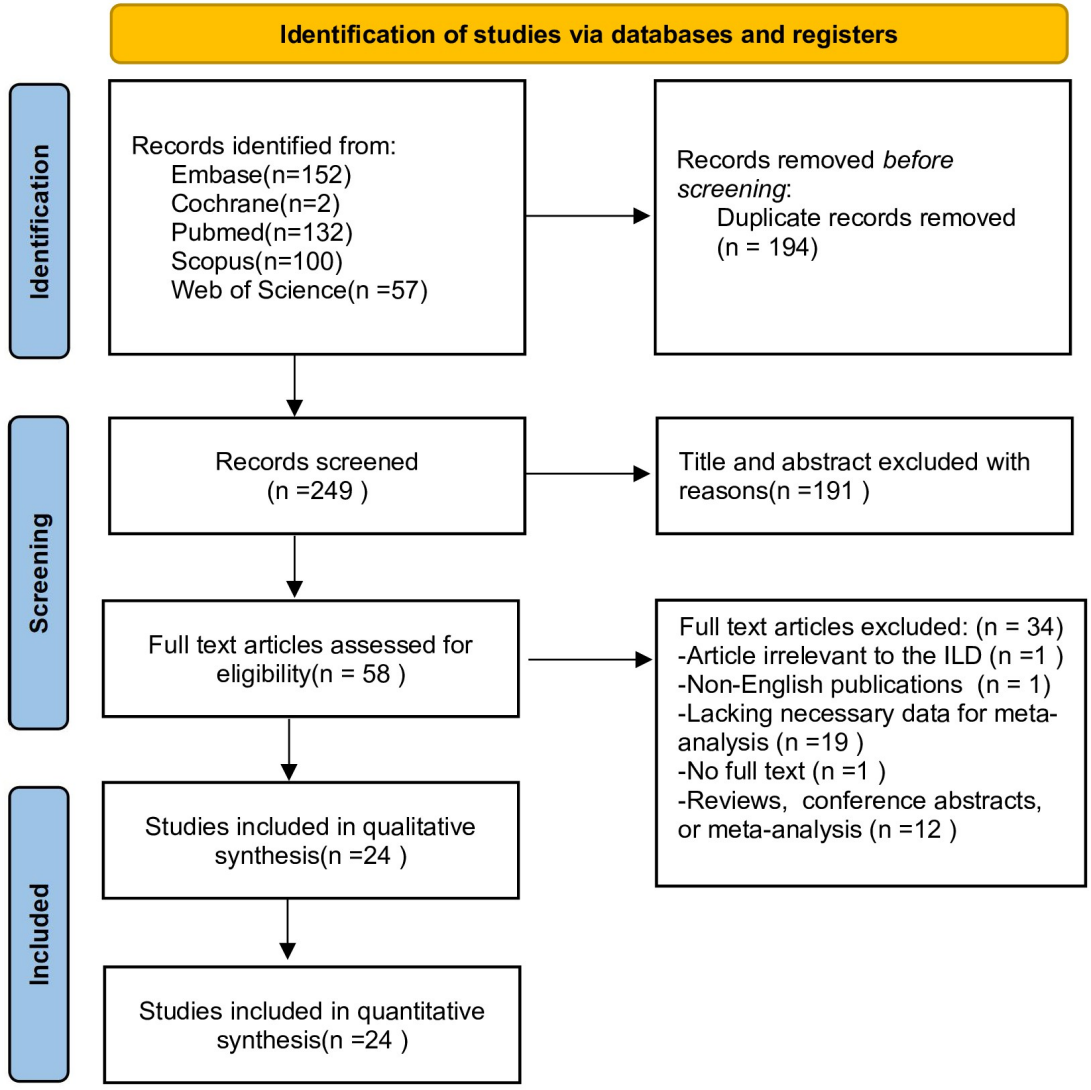
PRISMA flow diagram of the study selection.

The research selection flow chart is depicted in Fig 1. The database identified 443 articles for review. After removing duplicated literature, 249 articles were screened out, with 58 of them meeting the inclusion criteria. Of the 58 articles, one did not involve ILD, one was published in a language other than English, one did not provide the complete text, 12 were non-observational studies (conference abstracts, reviews, etc.), and 19 lacked the required data content for meta-analysis. This meta-analysis finally included 24 articles and 7,503 participants [5–22, 29–34].

### Study characteristics

The included research was published between 2015 and 2022. Of the 24 studies, eight were from China, two from Italy, two from Japan, three from South Korea, two from Romania, one from Saudi Arabia, one from Spain, three from the UK, and two from Turkey. Two studies were cross-sectional, 12 were retrospective case-control, and 10 were retrospective cohort studies. Adverse outcomes included mortality, worsening radiological lung interstitial change, progressive dyspnea and hypoxemia, and FVC decline ≥ 10% per year. Among the included studies, 15 articles (ILD:n = 2,912, Non-ILD: n = 2,868) compared NLR between patients with and without ILD [5, 8, 11, 13, 15, 17–22, 29, 31, 32, 34], and eight articles (favorable outcomes of ILD: n = 340, unfavorable outcomes of ILD: n = 407) compared NLR between ILD patients with favorable and unfavorable outcomes [6, 8–10, 15, 16, 32, 33] (Tables 1 and 2). The adjusted RR/HR/OR (95% CI) values of ILD patients with adverse outcomes were collected from nine studies [6, 7, 9–12, 30–32].

**Table 1:** Characteristics of included studies investigating patients with versus without ILD.

			With ILD				Without ILD				
First author, Country, Year	Study design	Disease	N.	Male (%)	Mean age	NLR (Mean ± SD)	N.	Male (%)	Mean age	NLR (Mean± SD)	NOS/AHRQ
Achaiah, UK., 2022 [31]	cohort	ILD	1259	602 (47.8)	65.39	3.92±5.22	355	203 (57.2)	63.4	4.46±5.79	8
Atilla, Turkey, 2016 [22]	case-control	SSc	34	9 (26.5)	NR	3.66 ± 1.32	25	6 (24)	NR	2.85 ± 1.12	8
Bai, China, 2021 [13]	cohort	IIM	133	44 (33.1)	50	5.7861±4.1821	153	47 (30.7)	47	5.5248±3.8169	9
Chen, China, 2019 [19]	case-control	RA	103	24 (23.3)	60.94	5.55±7.88	198	39 (19.7)	59.80	3.40±3.17	9
García-Núñez, Spain, 2022 [8]	case-control	silicosis	91	91 (100)	40.48	2.5252±1.2348	22	22 (100)	36.4	1.60±0.50	6
Ha, Korea, 2018 [34]	case-control	IIM	101	NR	NR	1.495± 0.761	96	NR	NR	1.225±0.902	7
Jung, Korea, 2017 [21]	case-control	SSc	40	NR	NR	6.13±9.18	48	NR	NR	2.12±1.71	7
Karataş, Turkey, 2019 [18]	case-control	silicosis	573	NR	40	3.295±4.9868	222	NR	35	2.2049±2.7982	8
Kim, Korea, 2020 [17]	cross-section	SSc	54	NR	NR	2.2455±1.272	60	NR	NR	1.6195±0.5469	8^†^
Man, Romania, 2022 [5]	case-control	ILD	36	20 (55.6)	64	2.7177±1.4978	161	33 (20.5)	40	2.0721±0.7481	6
Ruta, Romania, 2020 [15]	case-control	IPF/CTD	42	25 (59.5)	62.28	2.8229±1.1604	50	24 (48)	55.04	2±1.05	7
Xu, China, 2022 [29]	case-control	RA	284	105 (37.0)	68.95	4.37 ± 3.29	1215	274 (22.6)	57.95	3.84 ± 2.77	7
Yang, China, 2017 [20]	case-control	DM	18	9 (50)	51.39	5.54 ± 1.60	55	24 (43.6)	50.05	4.16 ± 1.73	8
Zhang, China, 2021 [11]	cohort	PSS	71	12 (16.9)	60.8	2.12±0.4	146	11 (7.5)	56.4	1.64±0.3	6
Zinellu, Italy, 2020 [14]	cross-section	IPF	73	22 (30.1)	69.64	2.3949±0.5899	62	19 (30.6)	67.35	1.9159±0.63	7^†^

^†^ The methodological quality of cross-sectional studies was evaluated by the Agency for Healthcare Research and Quality(AHRQ).

NLR,neutrophil-to-lymphocyte ratio;ILD,interstitial lung diseases;N., number; SD, standard deviation; NOS, the modified Newcastle-Ottawa Quality Assessment Scale; AHRQ, the Agency for Healthcare Research and Quality; NR, no report; SSc, systemic sclerosis; IIM, idiopathic inflammatory myopathy; RA, rheumatoid arthritis; IPF, idiopathic pulmonary fibrosis; CTD, connective tissue diseases; DM, dermatomyositis, PSS, primary Sjogren’s syndrome.

**Table 2:** Characteristics of included studies investigating ILD patients with versus without poor outcomes.

				With poor outcomes				Without poor outcomes					
First author, Country, Year	Study design	Disease	HR/RR/0R (95%CI)	N.	Male (%)	Mean age	NLR (Mean ± SD)	N.	Male (%)	Mean age	NLR (Mean ± SD)	Poor outcomes	NOS
Achaiah, U.K., 2022 [10]	cohort	IPF	1.31 (1.16–1.48)	53	39 (73.6)	74.4	3.1559±1.6155	62	49 (79.0)	74.8	2.6366±1.2751	FVC decline ≥10%/ year	8
Chen, China, 2022 [9]	cohort	IPF	1.019 (1.001–1.037)^‡^	161	127 (78.6)	68.45	8.18 ± 11.60	117	93 (79.5)	67.62	4.49 ± 4.76	Death	9
García-Núñez, Spain, 2022 [8]	case-control	silicosis	NR	38	38 (100)	41	2.7±1.5	53	53 (100)	40.1	2.4±1	Progress massive fibrosis	6
Paliogiannis, Italy. 2020 [16]	case-control	COVID-19	NR	9	NR	NR	15.545±16.0941	21	NR	NR	6.0354±7.2348	Death	7
Ruta, Romania, 2020 [15]	case-control	IPF/CTD	NR	6	NR	NR	2.3217±0.6576	36	NR	NR	2.9103±1.2149	Death	7
Shirakashi, Japan, 2020 [33]	cohort	DM	NR	13	5 (38.5)	62	8.5505±7.7242	25	6 (24)	45	3.7284±1.8867	Progress hypoxemia	6
So, China, 2022 [6]	cohort	DM	1.674 (0.710–3.948)	47	21 (44.7)	56.5	8.4±6.4	69	30 (43.5)	49.6	5.9±4.5	Progressive dyspnoea and radiological worsen	8
Achaiah, U.K., 2022 [31]	cohort	ILD	1.07 (1.05–1.09)	183	NR	NR	NR	1076	NR	NR	NR	Death	8
Mikolasch, U.K., 2022 [7]	cohort	IPF	2.04 (1.09–3.81)	533	NR	NR	NR	466	NR	NR	NR	Death or transplant	6
Saku, Japan, 2021 [12]	cohort	RA	1.010 (0.885–1.118)	22	NR	NR	NR	50	NR	NR	NR	Death	8
Touman, Saudi Arabia, 2022 [30]	case-control	COVID-19	5.928 (1.243–28.273)^‡^	19	14 (73.7)	59.74	NR	19	10 (52.6)	54.36	NR	Persistent radiological worsen	8
Zhang, China, 2021 [11]	cohort	PSS	1.43 (1.12–2.57)^§^	35	NR	NR	NR	182	NR	NR	NR	Death	6
Zou, China, 2015 [32]	cohort	DM	1.090 (0.956–1.242)	13	5 (38.5)	52	12.9745±6.7275	24	13 (54.2)	51	6.9818±7.6442	Death	8

^‡^OR

^§^RR

NLR,neutrophil-to-lymphocyte ratio;ILD,interstitial lung diseases;N.,number;SD,standard deviation;NOS,the modified Newcastle-Ottawa Quality Assessment Scale;NR,no report;OR,odds ratio;RR, relative risk;HR,hazard ratio;RA,rheumatoid arthritis;DM,dermatomyositis;IPF, idiopathic pulmonary fibrosis; CTD,connective tissue diseases;PSS,primary Sjogren’s syndrome;FVC,Forced vital capacity.

### Meta-analysis results

#### 1. Comparison of NLR between ILD and non-ILD patients

**Fig 2:**
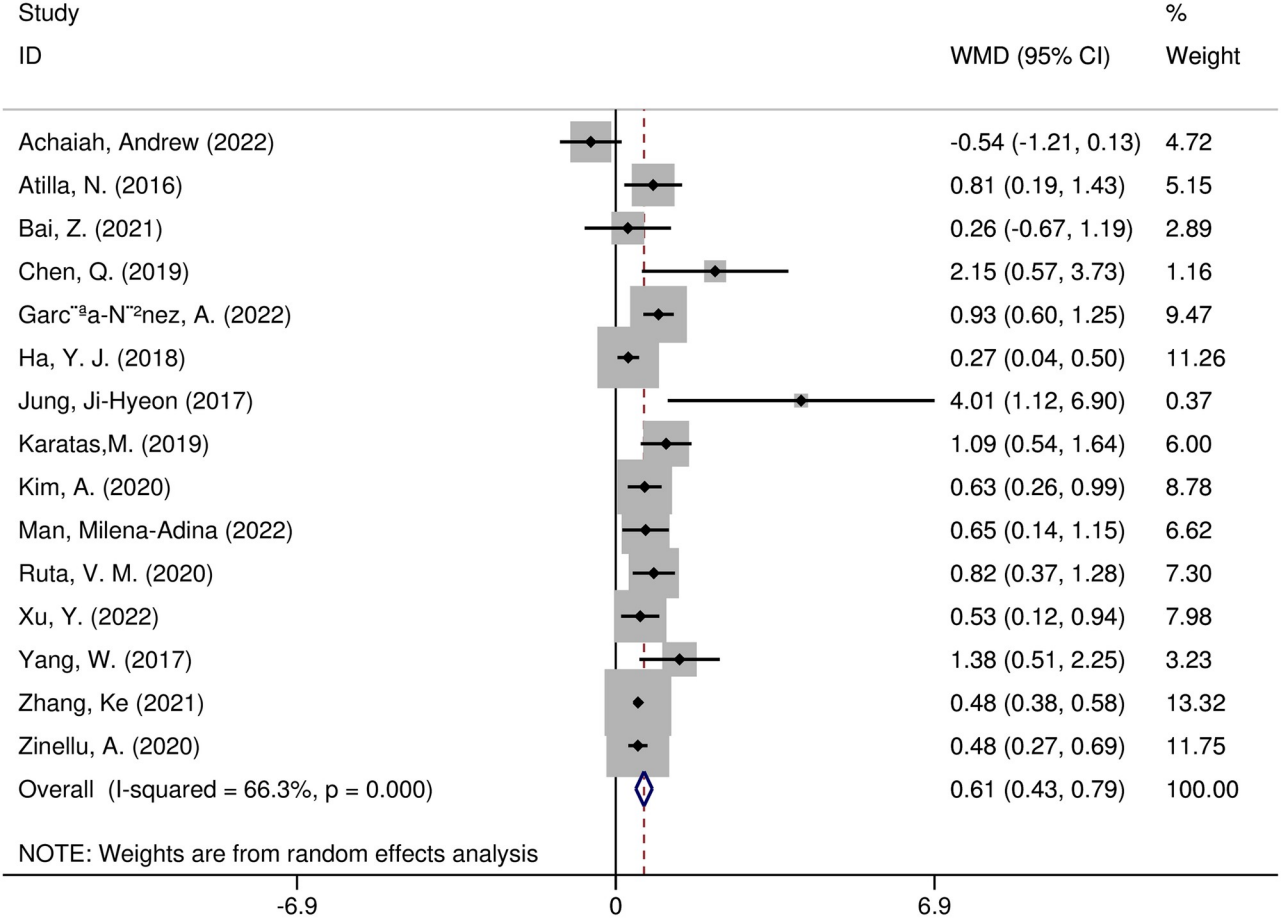
Forest plot illustrating the WMD (95% CI) of NLR levels between patients with and without ILD.

Fifteen studies compared NLR levels in patients with and without ILD [5, 8, 11, 13, 15, 17–22, 29, 31, 32, 34]. A random-effects model was utilized because the heterogeneity test showed statistical differences (I^2^ = 66.3%; *p* = 0.000). The NLR values were substantially increased in the ILD group (WMD = 0.61, 95% CI 0.43–0.79, p < 0.001) (Fig 2). Sensitivity analyses indicated that removing any single article from the meta-analysis did not affect the pooled WMD assessment. According to the analysis of the 15 articles included, the funnel plots of the two groups were roughly symmetrical (Begg test, *p* = 0.138; Egger test, *p* = 0.085) (S1 Fig). Overall, the findings were solid and consistent, showing that the NLR level in the ILD group was significantly higher than in the non-ILD group.

**Fig 3:**
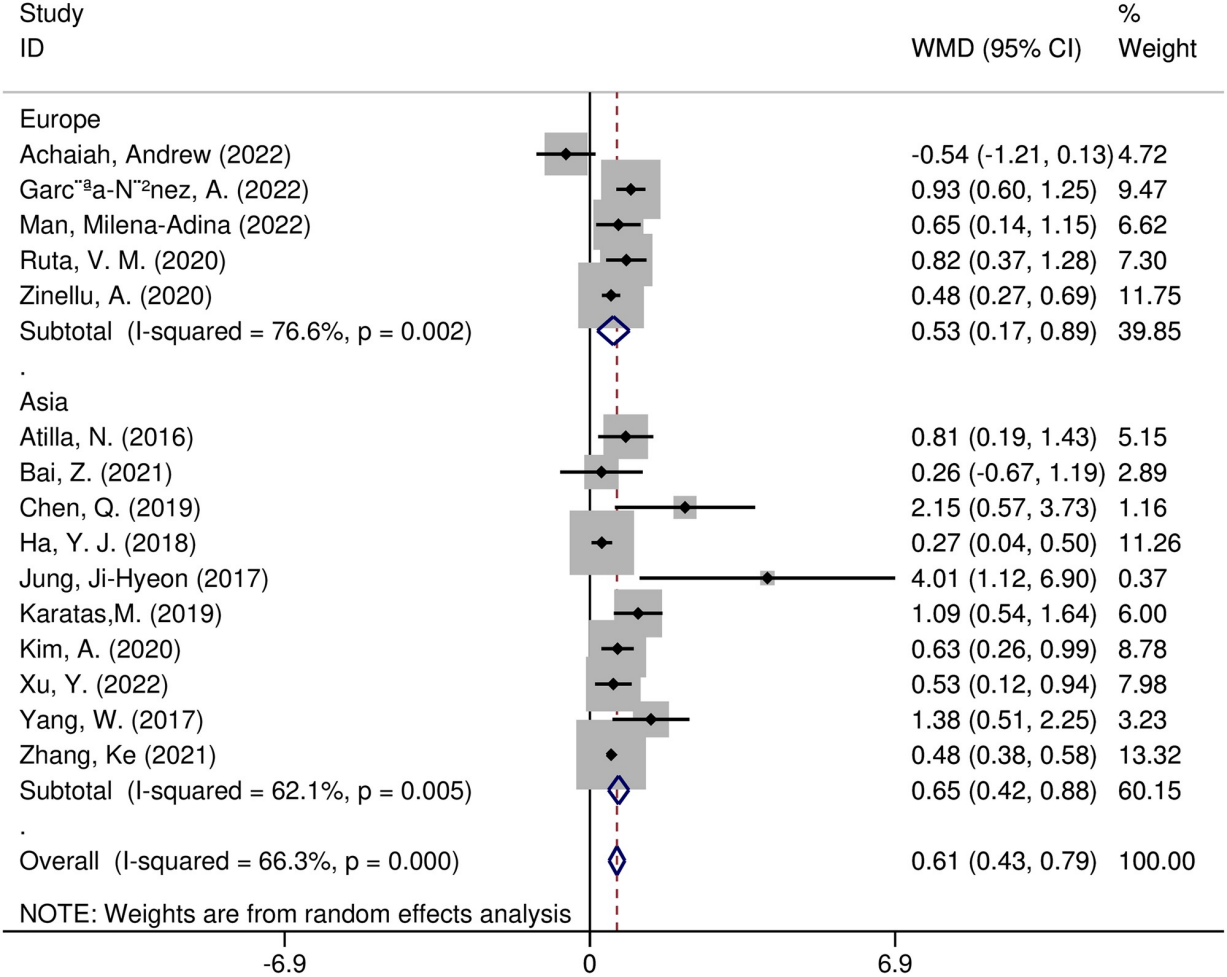
Forest plot illustrating the WMD (95% CI) of NLR levels between patients with and without ILD according to region.

**Fig 4:**
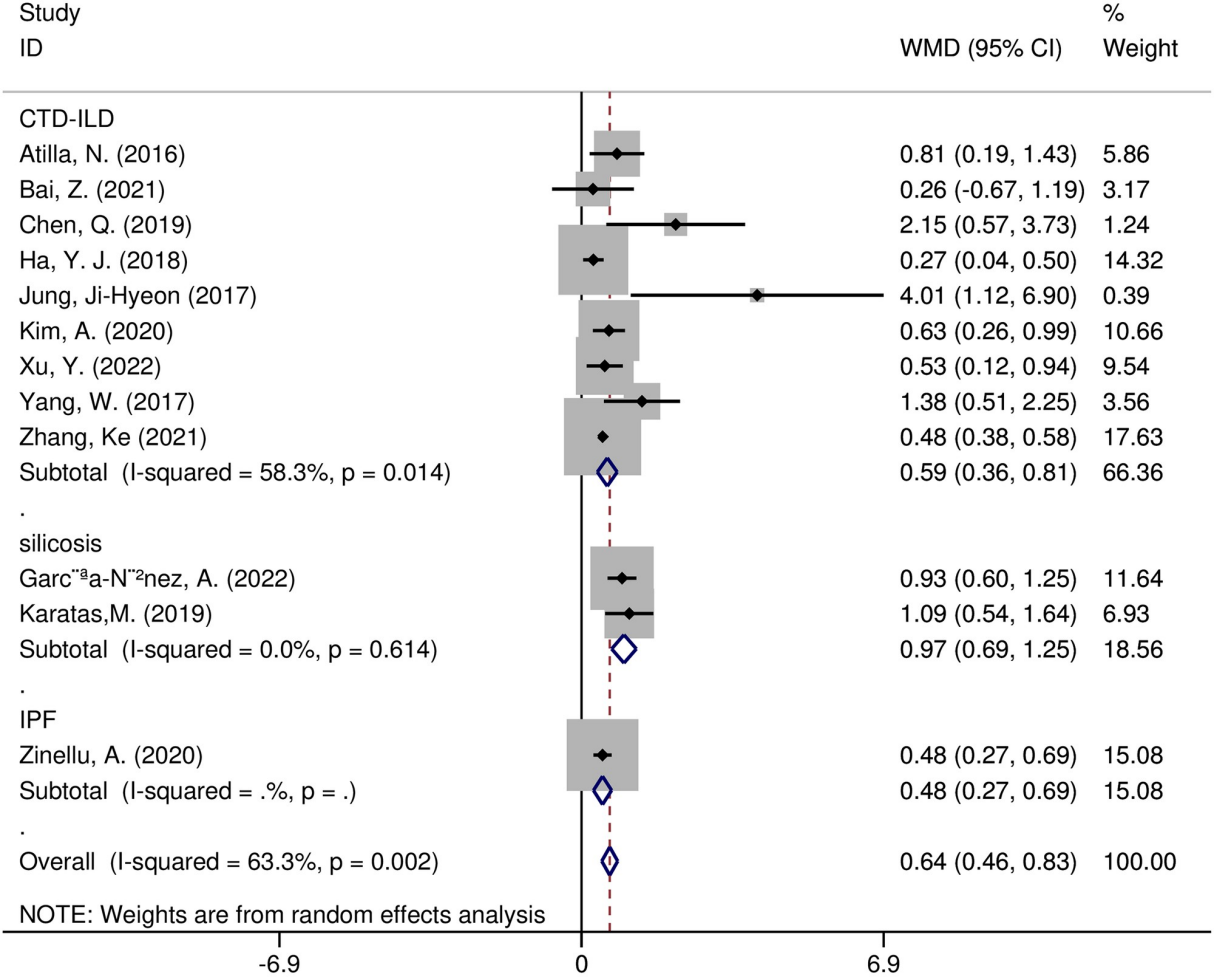
Forest plot illustrating the WMD (95% CI) of NLR levels between patients with and without ILD according to disease.

Furthermore, we performed meta-regression and subgroup analyses to detect potential sources of heterogeneity. According to the meta-regression analysis, the following variables were not sources of heterogeneity: region (t = 0.36, *p* = 0.728), size of the sample (t = 0.49, *p* = 0.634), study design (t = -1.53, *p* = 0.160), year of publication (t = -1.21, *p* = 0.256), or diseases (t = -0.60, *p* = 0.564). Several subgroup analyses were undertaken on region, study design, and diseases. The pooled effect size WMD was 0.65 (95% CI 0.42–0.88, *p* < 0.00001) in 10 Asian studies and 0.53 (95% CI 0.17–0.89, *p* = 0.004) in five European studies (Fig 3). The combined effect size for WMD was 0.59(95% CI 0.36–0.81, *p* < 0.0001) in the connective tissue disease associated with ILD(CTD-ILD) research, 0.97 (95% CI 0.69–1.25, *p* < 0.0001)in the silicosis research, and 0.48(95% CI 0.27–0.69, *p* < 0.0001) in the idiopathic pulmonary fibrosis(IPF) research (Fig 4). We found that the WMD was consistently greater in case-control studies (WMD = 0.82, 95% CI 0.53–1.12, *p* < 0.00001) and cross-sectional studies (WMD = 0.51, 95% CI 0.33–0.69, *p* < 0.00001), yet not in cohort studies (WMD = 0.11, 95% CI -0.58–0.79, *p* = 0.75) (S2 Fig). Except for the cohort studies, the other preset subgroup meta-analysis results indicated that NLR values in ILD patients were dramatically higher than those in the non-ILD group.

#### 2. Comparison of NLR between ILD patients with adverse outcomes and those without adverse outcomes

**Fig 5:**
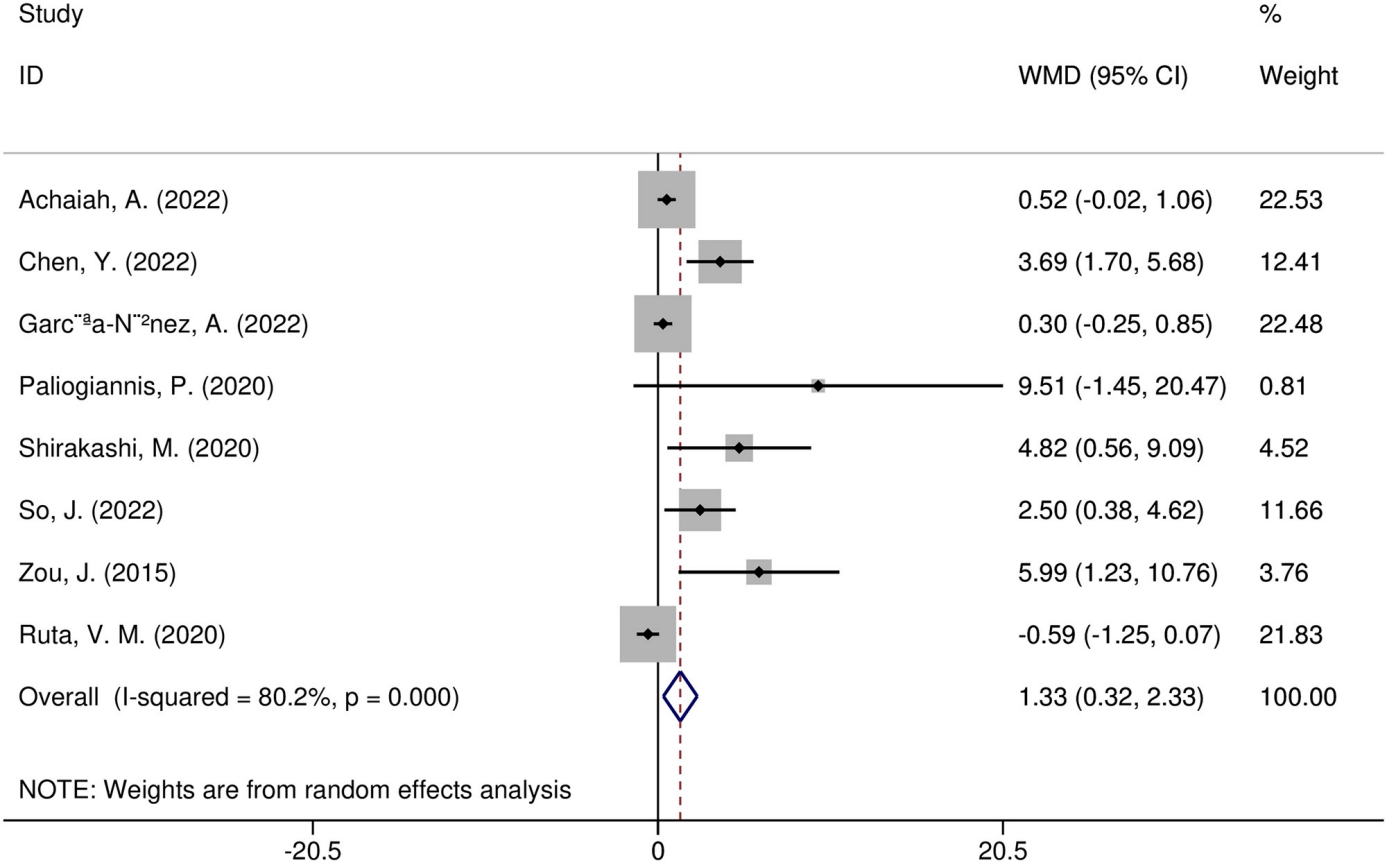
Forest plot illustrating the WMD (95% CI) of NLR levels between ILD patients with adverse and without adverse outcomes.

Eight studies compared the values of NLR between ILD sufferers with poor prognoses and those without poor prognoses [6, 8–10, 15, 16, 32, 33]. Adverse outcomes included death (four articles) [9, 15, 16, 32], radiographic deterioration (one article) [8], progressive hypoxemia (two articles) [6, 33], and FVC decline ≥ 10%/ year (one article) [10]. The included research indicated heterogeneity (I^2^ = 80.2%, *p* = 0.000), and the random effect model was utilized. NLR values were substantially greater in ILD patients with poor prognoses (WMD = 1.33, 95% CI 0.32–2.33, *p* = 0.01) (Fig 5). The effect sizes in sensitivity analyses matched those in the original analysis, although one study [9] altered the sensitivity analysis results, resulting in no statistically significant change. Because fewer than 10 included papers were included, an evaluation for publication bias was not completed.

**Fig 6:**
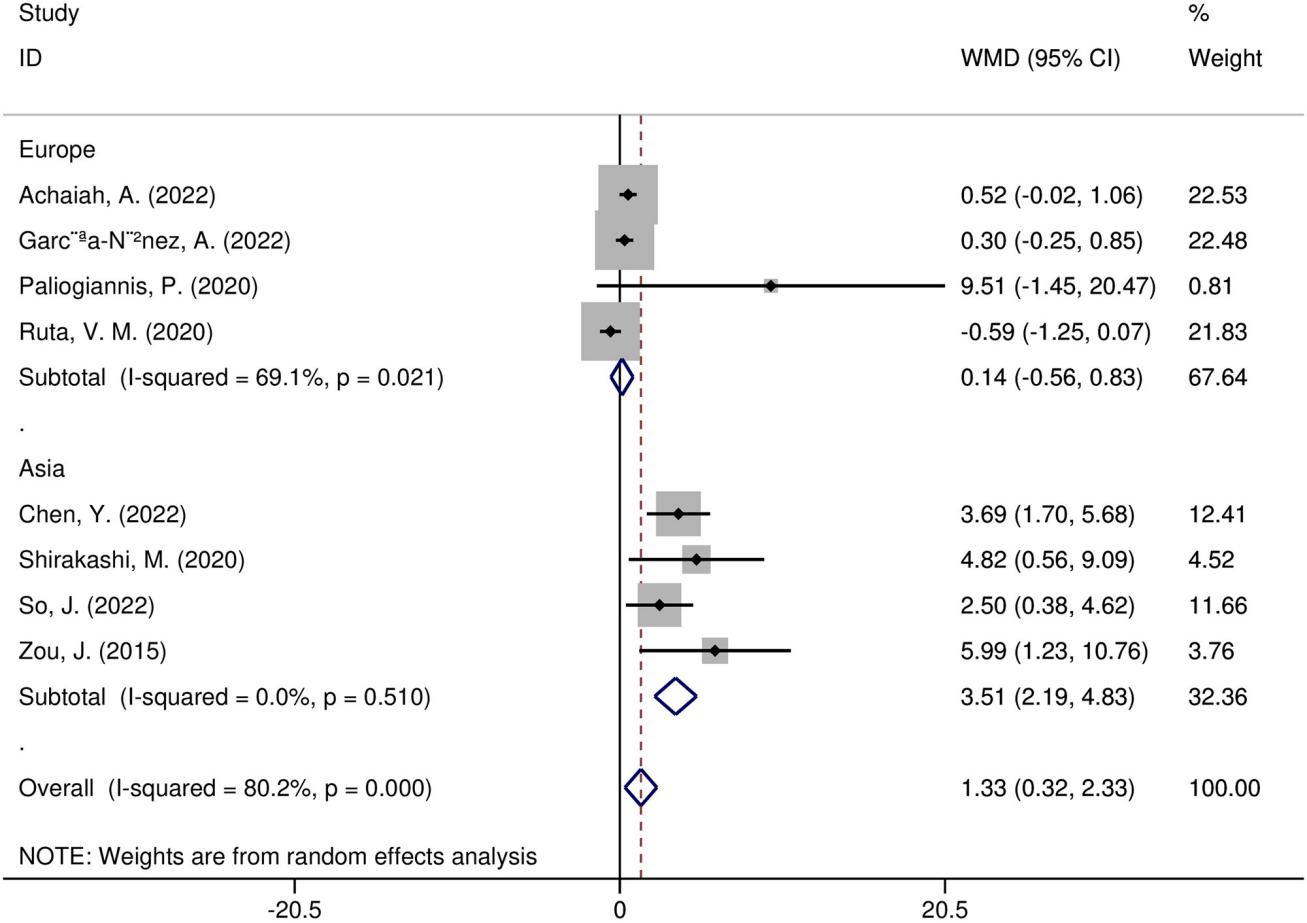
Forest plot illustrating the WMD (95% CI) of NLR levels between ILD patients with and without adverse outcomes according to region.

**Fig 7:**
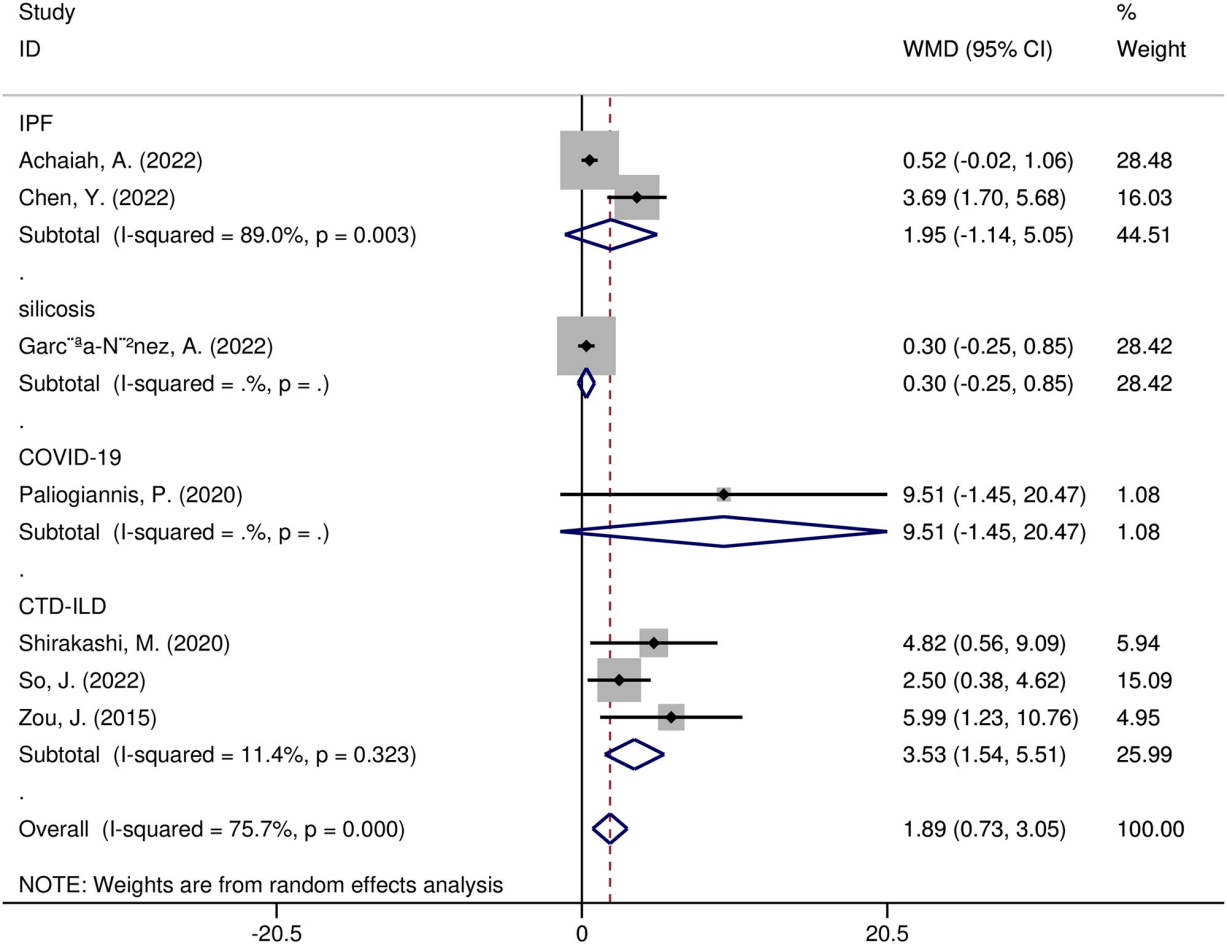
Forest plot illustrating the WMD (95% CI) of NLR levels between ILD patients with and without adverse outcomes according to disease.

**Fig 8:**
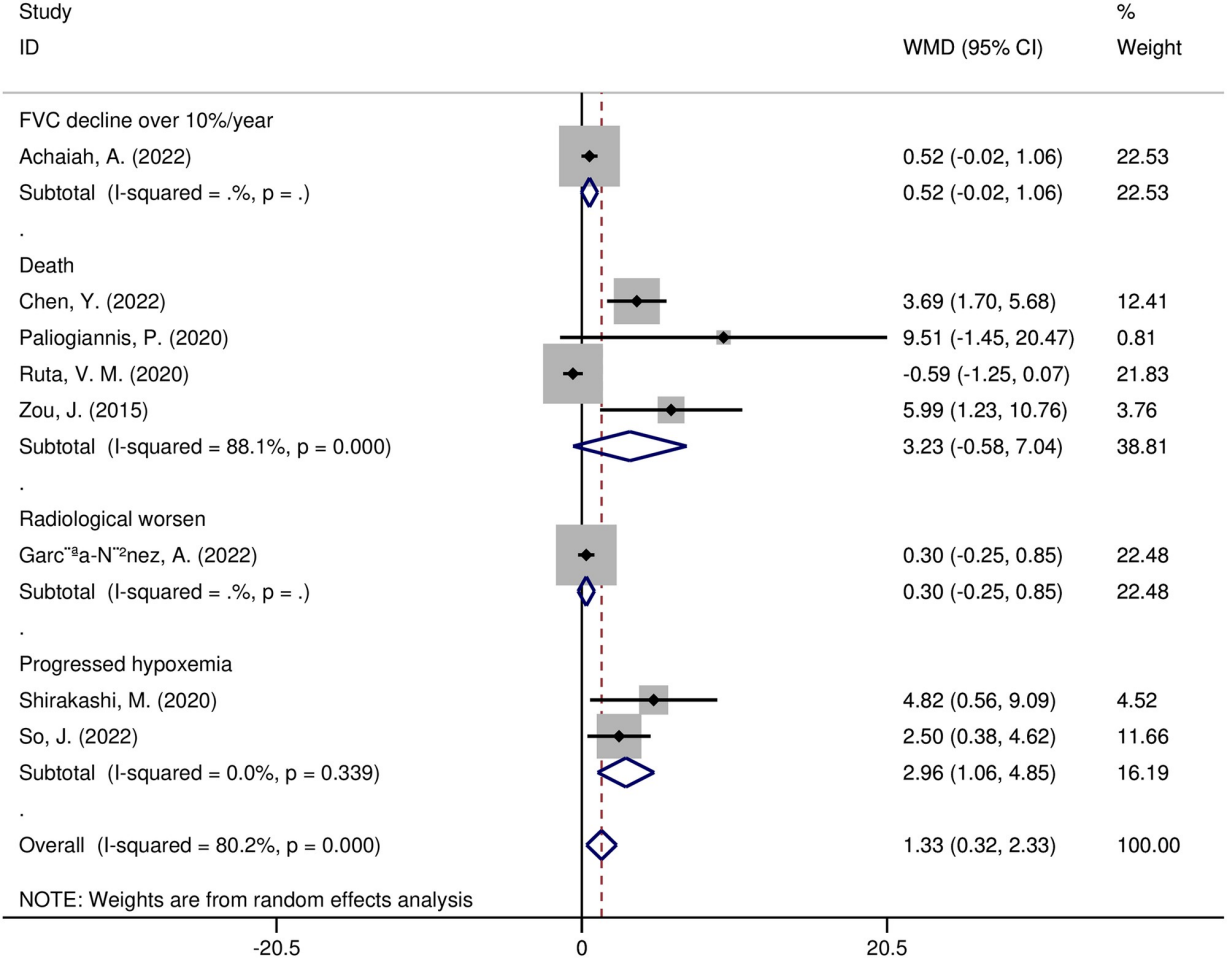
Forest plot illustrating the WMD (95% CI) of NLR levels between ILD patients with and without adverse outcomes according to outcomes.

In addition, meta-regression and subgroup analyses were carried out to investigate possible sources of heterogeneity. The following variables, according to the meta-regression analysis, were not sources of heterogeneity: region (t = -0.16, *p* = 0.898), sample size (t = 0.05, *p* = 0.966), study design (t = 0.08, *p* = 0.946), year of publication (t = -0.12, *p* = 0.921), diseases (t = 0, *p* = 0.999), or adverse outcomes (t = -0.13, *p* = 0.918). A subgroup analysis of the study regions showed that NLR was significantly higher in Asian research (WMD = 3.51, 95% CI 2.19–4.83, *p* < 0.00001), yet not in European research (WMD = 0.14, 95% CI -0.56–0.83, *p* = 0.70) (Fig 6). NLR values were substantially higher in the subgroup of CTD-ILD patients with adverse outcomes (WMD = 3.53, 95% CI 1.54–5.51, *p* = 0.0005), yet not in other disease subgroups (Fig 7). The NLR values increased in a subgroup analysis of adverse outcomes, including death, radiographic deterioration, progressive hypoxemia, and FVC decline≥10%/year. However, it only significantly increased in patients with progressive hypoxemia(WMD = 2.96, 95% CI 1.06–4.85, *p* = 0.002) (Fig 8).

**Fig 9:**
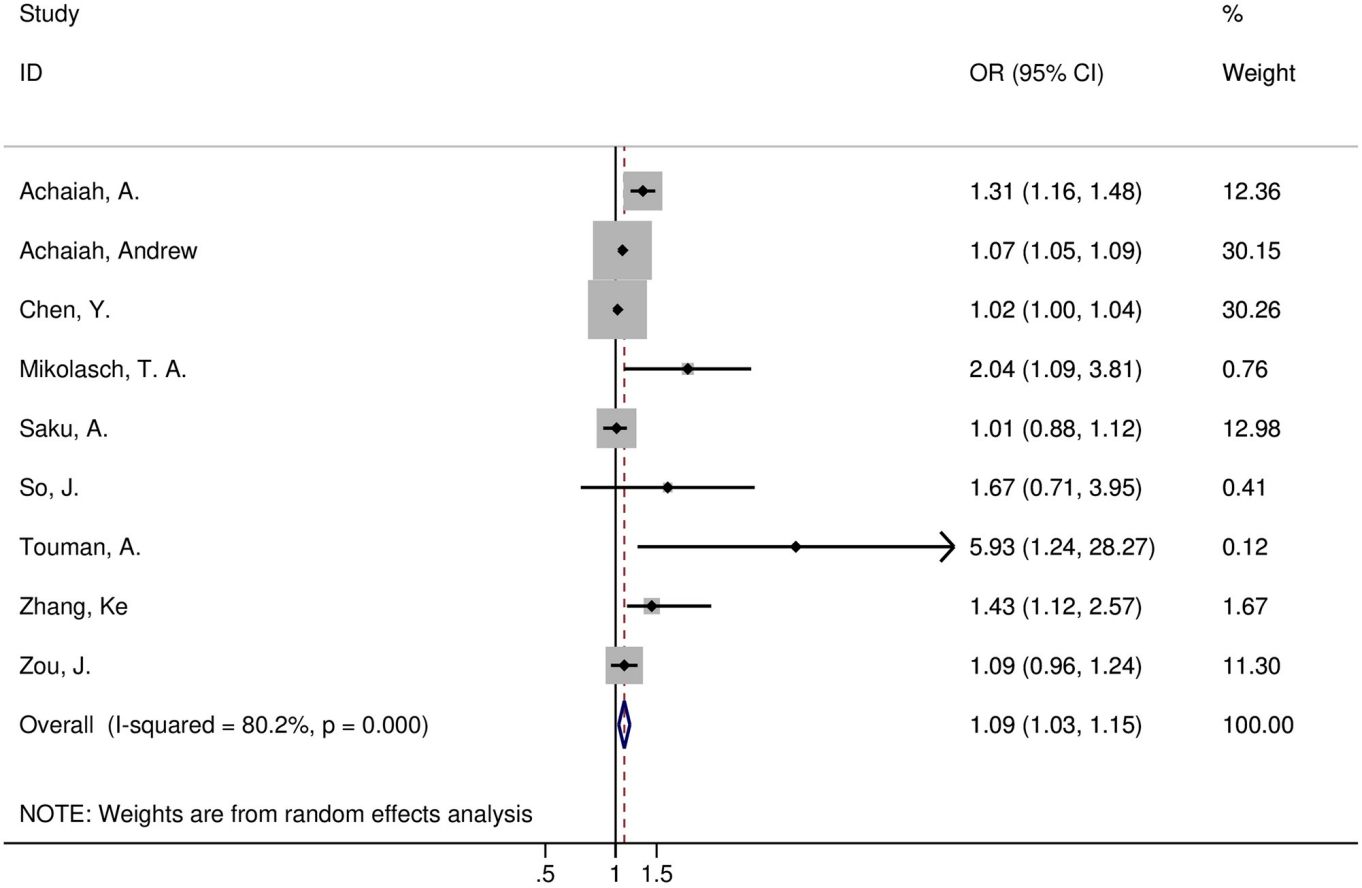
Forest plot illustrating that higher NLR levels were associated with adverse outcomes in ILD patients.

Nine articles [6, 7, 9–12, 30–32] evaluated the correlation between elevated NLR and adverse prognosis in ILD. Six studies [7, 9–11, 30, 31] found a link between high NLR levels and a poor prognosis, whereas three studies [6, 12, 32] found no association. The included literature exhibited apparent heterogeneity(I^2^ = 80.2%, *p* = 0.000), and a random effects model was used for statistical analysis. The meta-analysis showed a significant correlation between NLR and adverse prognosis in ILD patients(OR = 1.09, 95% CI 1.03–1.15, *p* = 0.0008) (Fig 9). Because fewer than ten studies were included, publication bias was not judged. Sensitivity analysis showed that the meta-analysis results remained stable after excluding the literature one by one. Therefore, we considered the elevated NLR value a risk factor for poor prognosis in ILD patients.

## Discussion

ILD is a group of diffuse lung parenchymal infiltrative diseases with different etiologies, mainly affecting the lung interstitium between the alveolar epithelial basement membrane and the capillaries. The lung interstitium is only a few micrometers thick in healthy individuals, containing lymphatic vessels, sporadic fibroblasts, and extracellular matrix proteins that promote effective gas exchange. The hallmark of ILD is inflammation or fibrosis within the interstitium, leading to impaired gas exchange, in many cases, resulting in respiratory distress, respiratory failure, and death.

The development of ILD involves both the natural and adaptive immune systems. Neutrophils are critical in the progression of ILD. Extracellular matrix (ECM) proteins can be degraded by neutrophil elastase and matrix metalloproteinases secreted by neutrophils; however, neutrophils can also promote ECM accumulation via the transforming growth factor-β (TGF-β) [3]. Neutrophils can form neutrophil extracellular traps (NETs), which can promote fibrosis through the production of TGF-β and activation of myofibroblasts, as well as exacerbate endothelial damage and infiltration of inflammatory cells in the lungs, leading to a vicious cycle of interstitial inflammation and fibrosis [35].

The function of lymphocytes is still debatable. Specific lymphocyte-secreted cytokines are thought to be profibrotic because they directly activate fibroblasts and myofibroblasts [36]. Th1 cells secrete interleukin (IL)-12 and interferon-γ (IFN-γ), which can alleviate fibrosis [37]. The classical Th2 cytokines, such as IL-4, IL-5, and IL-13, have been linked to fibrogenesis [38]. Several murine fibrosis model studies showed that the progression of pulmonary fibrosis could be slowed down by inhibiting IL-17A [39]. Tregs may play pro- and antifibrotic roles based on the degree of lung fibrosis and their interconnections with other T lymphocyte subgroups, which requires further investigation [40]. B cells have been found in higher concentrations in the lungs of patients suffering from IPF, rheumatoid arthritis (RA), and Sjögren’s syndrome, among other conditions [41, 42].

NLR results are more steady and comparable to absolute cell counts because factors such as dehydration, overhydration, and disparities in sample management can easily affect individual blood cell parameters. Recent studies have provided evidence that NLR is a vital biomarker of the inflammatory response, with a stronger predictive value for aggravation in many respiratory ailments [5]. Cortisol and epinephrine can increase neutrophil count while decreasing lymphocyte count, which could be the primary factors influencing NLR values. Other hormones and cytokines may also be involved. Lymphocytopenia reflects the intensity of stress and the immune system’s resistance and adaptability [43]. In severe trauma, major surgery, and exacerbating autoimmune diseases, circulating lymphocyte counts were significantly reduced [17, 43]. Patients with a higher NLR value have fewer lymphocytes but more neutrophils, which indirectly assesses the inflammatory status and cell-mediated immunity. Based on the above information, NLR levels in inflammatory diseases may be higher than in non-inflammatory diseases, and patients with serious illnesses may have higher levels than patients without serious illnesses [5].

The meta-analysis included 2912 ILD patients and 2868 non-ILD patients to explore the relationship between NLR levels and ILD. The pooled results suggested that NLR values were significantly elevated in the ILD group. Combined with the pathogenesis of ILD, it proved that NLR has particular value in predicting the occurrence of ILD. In clinical practice, if NLR is significantly raised after excluding infection in individuals with ILD risk factors, lung function and HRCT should be actively improved to identify whether the patient has ILD.

According to this study, NLR values were considerably higher in ILD patients with poor outcomes and thus functioned as a risk factor for those patients. Leveraging this biomarker may thus facilitate the early diagnosis and risk stratification of ILD. In the subgroup analysis, NLR had a higher value in predicting CTD-ILD prognosis compared to ILD produced by other causes(IPF, silicosis, COVID-19). When comparing the NLR levels of ILD sufferers with and without poor prognoses by region, the NLR values were significantly higher in ILD populations with poor prognoses in the Asian studies subgroup. This disparity might be because CTD-ILD was primarily included in the Asian literature. The mechanisms underlying the high NLR in CTD-ILD patients and its association with a worse prognosis in ILD patients remain unknown. We presume that this is a mark of continuing inflammation.

The pathogenesis of most ILD is characterized by inflammation or fibrosis or both, with initial inflammation progressing to fibrosis. IPF is the most common form of fibrotic ILD. In patients with IPF, fibrosis seems to depend on a triad of factors: lifelong epithelial over-injury caused by inhaled harmful substances, aging, and hereditary vulnerability [44]. Early senescence of alveolar epithelial stem cells leads to abnormal wound healing responses, antifibrotic and profibrotic mediators imbalanced, and ECM deposition, resulting in structural destruction of alveolar air sacs and airway remodeling. Changes in ECM composition and increased lung stiffness further contribute to fibrosis progression, becoming self-perpetuating [45]. Animal experiments have also shown that sustained fibrosis did not necessarily require accompanying inflammatory reactions [46].

Several etiologies can cause inflammation in ILD, the most prevalent of which is CTD [45]. The high inflammatory response was an essential feature of anti-melanoma differentiation-associated protein 5 (MDA5) positive ILD patients. NETs were detected in the lung tissue of idiopathic inflammatory myopathy (IIM) patients with ILD, and the peripheral blood NETs level was much higher than that of IIM patients [47]. In vitro experiments revealed that compared with IIM patients, the plasma of IIM-ILD patients could induce neutrophils to generate more NETs, and the activity of deoxyribonuclease I, which degraded NETs, was significantly lower [48]. In CTD-ILD, autoantibodies activated specific macrophages and matrix cells, releasing a series of cytokines, including anti-tumor necrosis factor(TNF), IL-1, IL-6, and prostaglandins, which promoted the progression of ILD. For example, RA-ILD was strongly correlated with high levels of rheumatoid factor and elevated levels of anti-citrullinated protein antibodies [45]. Positive expression of MDA5 was associated with rapid progression of ILD and high mortality in patients with DM.

Compared with IPF patients, SSc-ILD patients had more Th17 and Th22 cells in circulation [49], while RA-ILD patients had more B cells and CD4+ T cells in lung tissue than IPF patients, implying that CTD-ILD patients may be more prone to immune dysfunction than IPF patients [50]. Clinical trial results further confirmed that the pathogenesis of CTD-ILD differed from that of IPF. In several multi-center clinical trials, anti-inflammatory drugs (prednisone, TNF-α)or immunomodulators (IFNγ, simtuzumab) failed to improve lung function, control disease progression, or improve survival in IPF. They may even have a negative effect [51]. In contrast, CTD-ILD, characterized by inflammatory-driven factors, could usually benefit from anti-inflammatory and immunosuppressive treatments.

According to research, blood NLR levels were closely correlated with disease severity and mortality in patients with CTD-ILD [11, 13, 15, 17, 19, 34]. NRL was inversely linked with the carbon monoxide transfer coefficient in SSc-ILD patients and could be used as a sign of lung involvement [22]. Based on the above information and the results of this meta-analysis, we believe that NLR, as a new type of inflammatory indicator, has a more prominent role in predicting the poor prognosis of CTD-ILD.

There are still few effective treatments for ILD. Pirfenidone and nintedanib reduced FVC decline in IPF patients and increased survival. However, antifibrotic drugs are not curative, and lung functional stability alone may not protect against premature mortality in IPF [52]. Lung transplantation is the only way to extend life expectancy in the final stages of IPF. During treatment and follow-up, ILD patients face a substantial financial burden. Requiring individuals with high-risk factors for ILD or ILD patients to undergo HRCT screening every year may not be feasible in clinical practice. Though not a perfect test, NLR is a simple, cheap, repeatable, part of routine laboratory investigations and a non-invasive test compared to conventional assessment tools. NLR could be a compelling clue for the clinical detection of ILD, particularly in those at high risk of developing ILD (such as CTD patients and workers exposed to silica). If the NLR is significantly increased in such patients, HRCT for ILD screening should be performed. Furthermore, the NLR is appropriate for long-term follow-up of ILD patients. Observing the continuous change in NLR values in ILD patients can provide helpful information about patients’ physiological pressure and disease statuses.

The well-designed meta-analysis methodology, combined with the inclusion of all eligible studies, provided sufficient statistical power to reach a broad conclusion. The meta-analysis had some limitations. First, there was significant heterogeneity, and no source of heterogeneity was discovered despite using subgroup and meta-regression analyses. Differences in ILD etiology and poor prognosis outcome criteria across studies might explain this heterogeneity. Second, retrospective studies were included, and some outcome indicators had a small number of included studies. Third, no medication treatment options were provided in the included literature, and drugs that affect the NLR cannot be ruled out completely. Fourth, studies from the Americas and Africa were scarce. Fifth, we only included articles in English, potentially resulting in bias.

## Conclusions

In conclusion, our findings demonstrated that the increased NLR values were significantly linked to the possibility of ILD occurrence and poor outcomes, especially in CTD patients. Given this study’s limited research, prospective studies with larger sample sizes, long-term follow-up, and multi-center studies are required.

## Supporting information

S1 Fig Funnel plot of studies investigating NLR in patients with and without ILD. (TIF)

S2 Fig Forest plot illustrating the WMD (95% CI) of NLR levels between patients with and without ILD according to the study design. (TIF)

S1 Checklist PRISMA checklist. (PDF)
