# Supplementary figures and images for: A meta-analysis of the clinical significance of neutrophil-to-lymphocyte ratios in interstitial lung disease
Source: PLoS One. 2023 Jun 12;18(6):e0286956. doi: 10.1371/journal.pone.0286956 (PMC10259798; doi:10.1371/journal.pone.0286956)

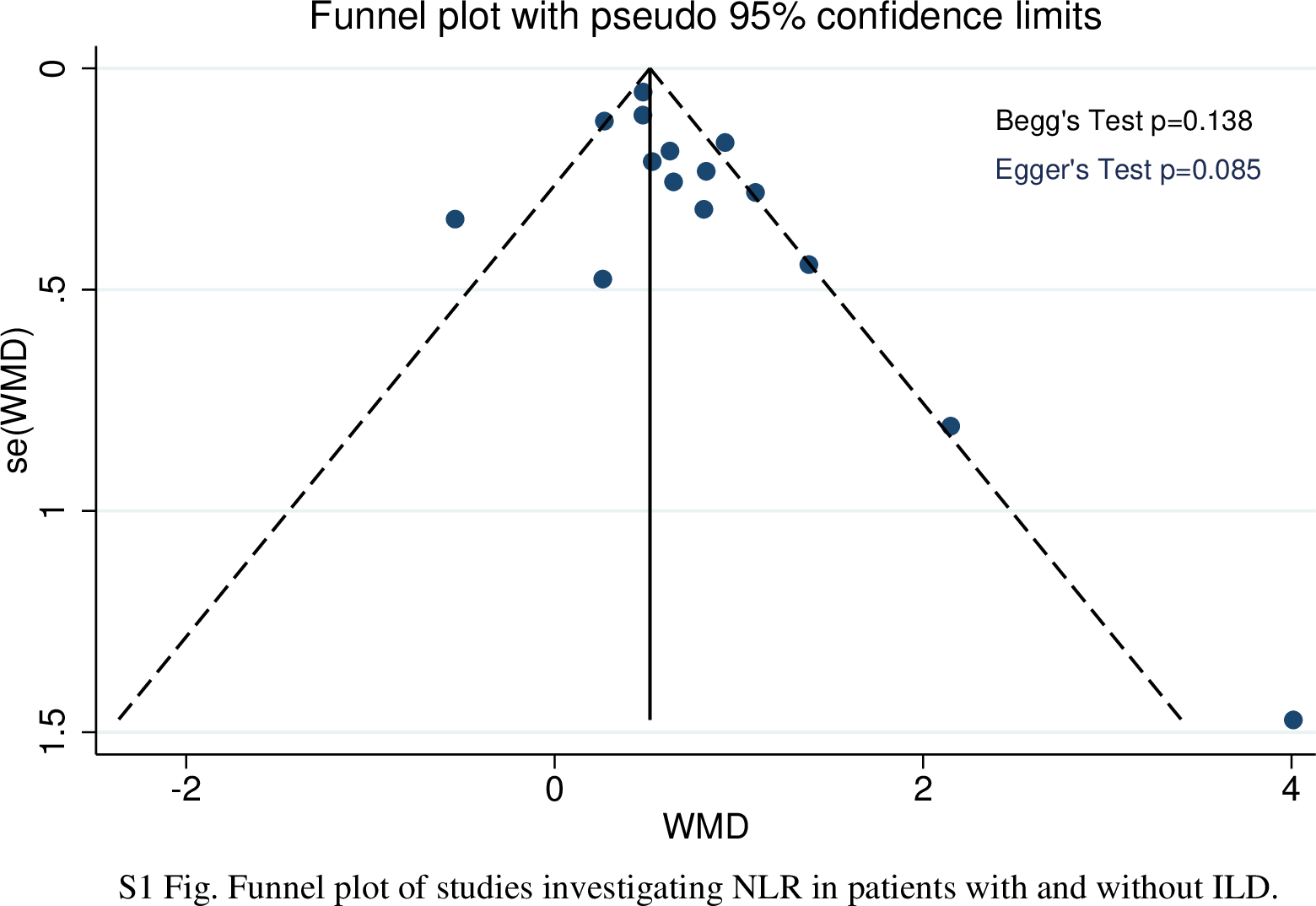

Supplement: S1 Fig — (TIF) [file pone.0286956.s001.tif]

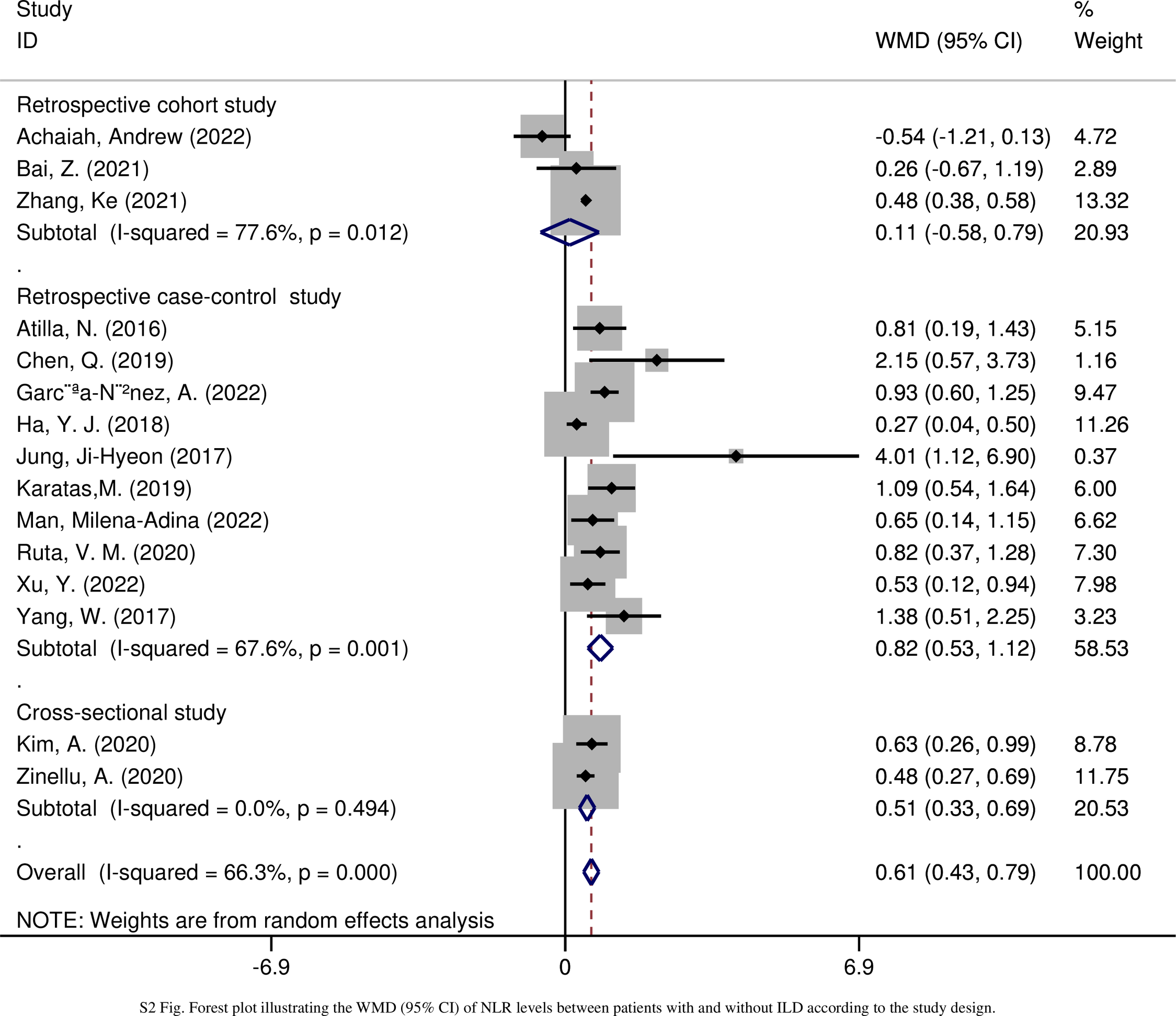

Supplement: S2 Fig — (TIF) [file pone.0286956.s002.tif]
